# Editorial: Nutrition and health-related quality of life: is it an ignored outcome? Volume II

**DOI:** 10.3389/fnut.2023.1213059

**Published:** 2023-05-30

**Authors:** Leila Itani, Emilia Vassilopoulou, Rosa Sammarco, Marwan El Ghoch

**Affiliations:** ^1^Department of Nutrition and Dietetics, Faculty of Health Sciences, Beirut Arab University, Beirut, Lebanon; ^2^Department of Nutritional Sciences and Dietetics, International Hellenic University, Thessaloniki, Greece; ^3^Internal Medicine and Clinical Nutrition Unit, Department of Clinical Medicine and Surgery, Federico II University Hospital, Naples, Italy; ^4^Faculty of Medicine, UniCamillus—Saint Camillus International University of Health and Medical Sciences, Rome, Italy

**Keywords:** nutrition, outcome, obesity, health related quality of life, handgrip

In recent years, there has been a growing interest in Health-Related Quality of Life (HRQoL) (1), defined as an individual's or a group's perceived physical and mental health over time (2). HRQoL is frequently assessed alongside medical and psychological outcomes in many clinical settings and public health services, across a wide spectrum of diseases (3) and is considered an important dimension to measure during the development of new treatments (4, 5).

Since the Ancient Greek era, the impact of nutrition on health has been widely reported (6, 7), yet there remains a lack of knowledge about the link between nutrition and HRQoL (8). Our Research Topic, entitled “*Nutrition and health-related quality of life: is it an ignored outcome? Volume II*,” aimed to attract research from diverse backgrounds focusing on both human nutrition and HRQoL. We were particularly interested in work that may clarify the link between human nutrition and HRQoL, and the nature of their interaction. We received five submissions; two were rejected and three original research papers were accepted following peer review. The submissions are international, from America and Europe.

In the first study, conducted in America, Han et al. considered grip strength as a valid indicator of HRQoL in a study with 2,127 participants of both genders aged 60 years and above (9). They evaluated the association between dietary magnesium intake and handgrip strength, and whether this association was influenced by serum vitamin D status. They found that low magnesium intake was associated with reduced handgrip strength in participants with a deficient serum concentration of 25(OH)D. They concluded that there is a need to increase magnesium intake in people with this deficiency in order to maintain suitable muscle strength and good HRQoL.

In the second study, conducted in Spain, de Lourdes Moreno et al. validated a Spanish language version of the Coeliac Disease Questionnaire (CDQ). This simple instrument is widely used to assess HRQoL in patients with coeliac disease (10) and this work will enable better assessment of HRQoL in the Spanish population.

In the third study, also from Spain, Alonso-Cabezas et al. investigated adherence to a healthy diet, considered to be the basis of good HRQoL, in 1,251 premenopausal women aged between 39 and 50. Perhaps surprisingly, only a third of the participants demonstrated adequate adherence to the specific dietary recommendations for this population. HRQoL was not directly and objectively measured in this study and therefore may require further investigation.

All the studies included in this Research Topic either directly or indirectly explored the link between nutrition and HRQoL, which offers progress toward understanding their interaction. One study validated a useful tool to directly assess HRQoL and another identified the association between dietary intake, serum levels of certain micronutrients and HRQoL. Most notably, magnesium dietary intake and serum vitamin D levels can be considered important makers of reduced handgrip strength, which is an indirect measure of HRQoL. The last study underlined the importance of adhering to a healthy diet and maintaining normal weight in order to experience better HRQoL. Further research is required replicate and consolidate these findings, while considering the potential methodological issues in measuring HRQoL (11).

We are grateful to *Frontiers in Nutrition* for giving us the opportunity to serve as editors for this Research Topic. It has been a challenging, educational and motivating experience. We would like to thank the authors for sharing their research in this Research Topic, which we believe will be of particular relevance for a clinical readership. Finally, we would like to thank the reviewers for their time and input, which undoubtedly improved the quality of the studies published in this Research Topic.

## Author contributions

All authors claim authorship and have approved and made substantial contributions to the conception, drafting, and final version of the paper.
